# Real-time analysis of osteoclast resorption and fusion dynamics in response to bone resorption inhibitors

**DOI:** 10.1038/s41598-024-57526-9

**Published:** 2024-03-28

**Authors:** Preety Panwar, Jacob Bastholm Olesen, Galia Blum, Jean-Marie Delaisse, Kent Søe, Dieter Brömme

**Affiliations:** 1 Department of Biochemistry and Molecular Biology, Faculty of Medicine, The University of British Columbia, Vancouver, BC Canada; 2 Department of Oral Biological and Medical Sciences, Faculty of Dentistry, University of British Columbia, 2350 Health Sciences Mall, Vancouver, BC V6T 1Z3 Canada; 3 Clinical Cell Biology, Pathology Research Unit, Department of Clinical Research, University of Southern Denmark, Campusvej 55, 5230 Odense M, Denmark; 4 Department of Pathology, Odense University Hospital, Odense, Denmark; 5 Faculty of Medicine, Campus Ein Karem, The School of Pharmacy, Institute of Drug Research, The Hebrew University of Jerusalem, Room 407, 9112001 Jerusalem, Israel; 6 Department of Molecular Medicine, University of Southern Denmark, Odense, Denmark; 7 Department of Pharmaceutical Sciences, Elizabeth City State University, Elizabeth City, NC USA

**Keywords:** Human osteoclast, Live-imaging, Cathepsin K, Bone resorption, Active-site probe, Cell fusion, Biochemistry, Cell biology, Drug discovery, Diseases

## Abstract

Cathepsin K (CatK), an essential collagenase in osteoclasts (OCs), is a potential therapeutic target for the treatment of osteoporosis. Using live-cell imaging, we monitored the bone resorptive behaviour of OCs during dose-dependent inhibition of CatK by an ectosteric (Tanshinone IIA sulfonate) and an active site inhibitor (odanacatib). CatK inhibition caused drastic reductions in the overall resorption speed of OCs. At IC_50_ CatK-inhibitor concentration, OCs reduced about 40% of their trench-forming capacity and at fourfold IC_50_ concentrations, a > 95% reduction was observed. The majority of CatK-inhibited OCs (~ 75%) were involved in resorption-migration-resorption episodes forming adjacent pits, while ~ 25% were stagnating OCs which remained associated with the same excavation. We also observed fusions of OCs during the resorption process both in control and inhibitor-treated conditions, which increased their resorption speeds by 30–50%. Inhibitor IC_50_-concentrations increased OC-fusion by twofold. Nevertheless, more fusion could not counterweigh the overall loss of resorption activity by inhibitors. Using an activity-based probe, we demonstrated the presence of active CatK at the resorbing front in pits and trenches. In conclusion, our data document how OCs respond to CatK-inhibition with respect to movement, bone resorption activity, and their attempt to compensate for inhibition by activating fusion.

## Introduction

Bone resorption is essential for adapting bone structure to physiological demands, for renewing the bone matrix, and for adjusting calcemia^1^. Excessive resorption leads to bone loss and is a major component of pathological situations such as osteoporosis and arthritis^2,3^. Osteoclasts (OCs) originating from hematopoietic precursors and that become multinucleated through cell–cell fusion are responsible for bone resorption^4,5^. The actual resorption apparatus of the OCs is the ruffled border, a specialized area of the plasma membrane where resorption agents are secreted and where resorption products are taken up^6^. The ruffled border is surrounded by an actin ring that orients this ruffled border against the bone matrix. OC-mediated bone resorption can be divided into two critical steps: (1) demineralization by acidification catalysed by vacuolar-type ATPase (V-ATPase) and (2) organic matrix degradation by the predominantly expressed collagenase, cathepsin K (CatK)^7–11^. The critical role of CatK in osteoclastic bone resorption has been the basis for the development of active site-directed CatK inhibitors (e.g., odanacatib and balicatib) that prevented bone loss in osteoporotic patients, but also displayed side effects—possibly due to blocking the activity of CatK on non-collagen substrates^12^. It is, therefore, of great interest that tanshinones, extracted from the medicinal plant *Salvia miltiorrhiza*, were found to specifically block the collagenolytic activity of CatK without blocking its overall activity^13,14^. The collagenase activity of CatK requires the oligomerization of the protease in the presence of glycosaminoglycans, which is likely needed for the partial unfolding of triple helical domains within collagens prior to their cleavage^15–18^. In contrast, monomeric CatK retains its general proteolytic activity, but is unable to degrade triple helical collagens^16,18^.Thus, the prevention of CatK oligomerization by so-called “ectosteric inhibitors” selectively blocks the enzyme’s collagenase activity. We have previously demonstrated that these ectosteric inhibitors hinder bone resorption in preclinical models just as active site-directed inhibitors do^14,19^.

It should be emphasized that the level of active CatK significantly determines the propensity of OCs to make long resorption trenches (i.e., resorption parallel to the bone surface) rather than circular pits (resorption perpendicular to the bone surface)^20–23^. CatK levels vary amongst individuals^20,21^ and increase with age, menopause, and glucocorticoid treatment^24–26^. This may favor the more aggressive trench resorption mode. Recently, pit and trench forming OCs have been investigated through time-lapse approaches^27^ indicating the high mobility of resorbing OCs^24^. Thus, OCs can move and erode the bone surface at the same time, which then results in resorption cavities that appear as trenches^27^. However, a systematic time-lapse investigation of the effect of CatK inhibitors on these activities is still lacking, and the distribution of active CatK in OCs resorbing in trench mode remains elusive.

Another prerequisite for the resorptive activity of OCs is multinucleation. Multinucleation is often regarded as the final step of osteoclastogenesis and directly influences the resorptive activity of OCs^28–30^. Important for our present study were observations about the relationship between the number of nuclei per OC and CatK levels^31^ and that collagen-osteoclast interactions influence cell–cell fusion^32–34^. Interestingly, recent knowledge on the regulation of nuclearity has progressed significantly because of time-lapse approaches, which allows to distinguish fusion between multi- and mono-nucleated cells, and to take into account fission and specific characteristics of the fusion partners, such as their motility^21,35,36^. However, the effect of inhibiting CatK activity on OC fusion was never analyzed.

Using time-lapse approaches, the present study investigates the effect of CatK inhibitors on the movement, resorptive activity, and fusion of OCs while resorbing. Both the active site-directed inhibitor odanacatib (ODN) and the ectosteric inhibitor Tanshinone IIA sulfonate (T06) revealed comparable effects on the resorption activity of OCs indicating that the collagenase activity of CatK alone is the major proteolytic activity involved in bone resorption. Furthermore, we used a fluorescently-labeled active site CatK probe to localize active CatK in pit- and trench-forming OCs.

## Results

The progression of OC resorption and the dependency of migration from two mechanistically different CatK inhibitors (ODN and T06) was followed with a time-lapse approach using bovine cortical bone slices. F-actin (in green) and collagen staining (in red) were used to monitor the OC activity. Actin staining allows to visualize the sealing zone (SZ) and thus it’s associated ruffled border in OCs making a pit (annular-shaped; stationary resorption) or a trench (crescent-shaped; mobile resorption). No SZ means that the resorption apparatus is not activated. Rhodamine staining allows for visualization of the upper collagen layer of the bone matrix and its removal^27^. This allowed us to subcategorize the resorption events according to their duration (h), the prevalence of pits and trenches (%), the enlargement speed of pit diameter and trench length (µm/h), and the enlargement speed of their surface area, herein called trench resorption speed or pit formation speed (µm^2^/h), as well as their involvement in cell–cell fusion.

### The effect of CatK inhibitors on bone-resorbing OCs as visualized by time-lapse microscopy

**Figure 1:**
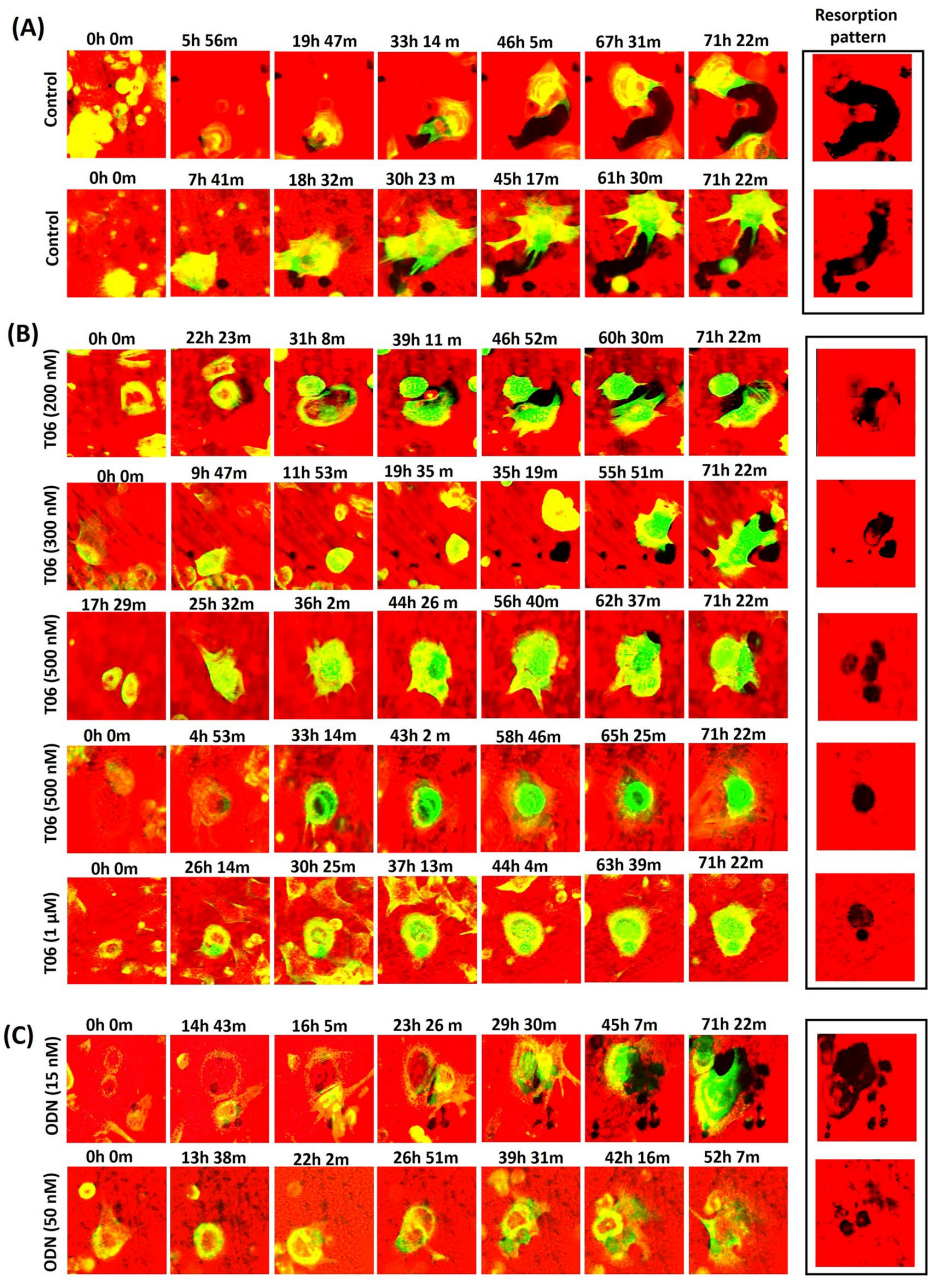
Selected time-lapse images of pit- and trench-forming OCs. Time-lapse images were taken from videos of control, T06—(ectosteric inhibitor), and ODN—(active site-directed inhibitor) treated OC cultures at different inhibitor concentrations (Video 1). (**A**) Selected OC images at various time points over 72 h showing OCs making a trench in the absence of a CatK inhibitor. The upper panel shows an OC starting in pit mode and subsequently switching into trench mode. The lower panel shows an OC first making a pit and then moving to a nearby area to generate a trench (Example 2, Video 1). (**B**) Selected OC images at various time points over 72 h showing OCs involved in pit and trench formation events in the presence of increasing T06 concentrations (200 nM, 300 nM, 500 nM, and 1 µM). (**C**) Selected time points of a 72 h OC culture in the presence of increasing concentrations of ODN (15 nM and 50 nM). Pit- and trench-forming OCs were stained for actin by using phalloidin (green), and the bone surface was stained with rhodamine (red). Resorption of bone surface by OCs is shown by black trench/pit imprints.

**Figure 2:**
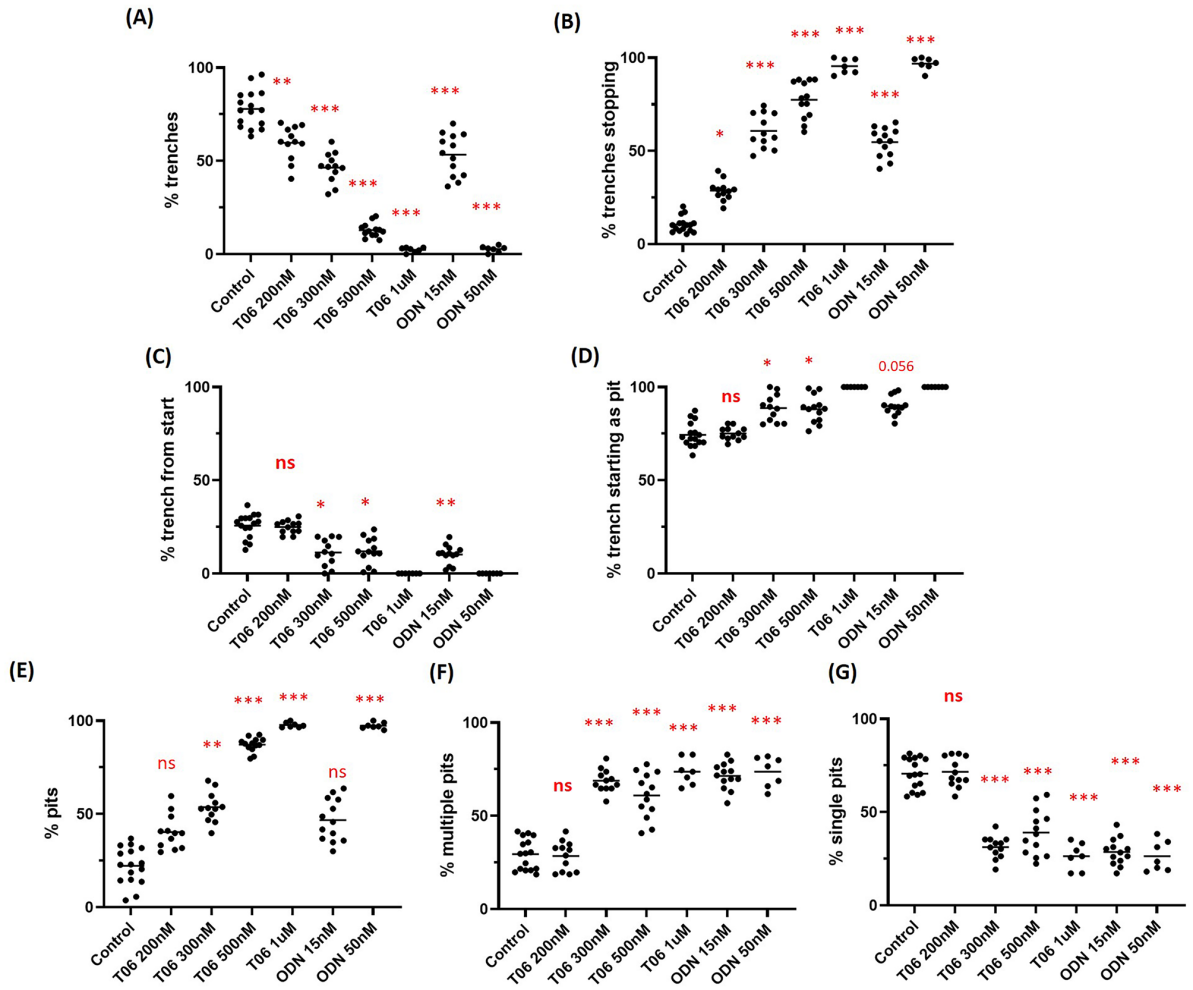
Prevalence of trench- and pit-formation events in the absence or presence of an ectosteric (T06) or active site-directed (ODN) CatK inhibitor over a period of 72 h (time-lapse). (**A**,**B**) The proportion of OCs in trench mode as a total percentage, and proportion of trench-making OCs stopping their elongation. (**C**,**D**) The proportion of resorption events starting either as a pit or directly as a trench. (**E**–**G**) The proportion of OCs in pit mode either starting as single or multiple pits. All are shown as total percentage. These proportions were assessed in independent experiments. Sample size: Control: n = 16 from 5 donors; 200 nM T06: n = 12 (3 donors); 300 nM T06: n = 12 (3 donors); 500 nM T06: n = 13 (4 donors); 1 µM T06: n = 7 (2 donors); 15 nM ODN: n = 12 (3 donors); 50 nM ODN: n = 7 (2 donors). For each donor, 3 to 5 replicate experiments on individual bone slices were analyzed for all conditions. We analyzed between 50 and 150 OCs per condition per experiment for each of the donors. The median proportions obtained in each experiment are shown as bars. Statistics: Kruskal–Wallis test, two tailed, Dunn’s multiple comparisons test compared groups with control verified significance difference. The * indicated in these graphs are based on the values obtained as per Kruskal; Dunn’s tests and summarized accordingly and represented as (ns, Not significant; *P < 0.05; **P < 0.01; ***P < 0.0001).

Both CatK inhibitors (T06 and ODN) changed the action of OCs from a predominant trench mode into a pit mode. Trench-forming OCs under non-inhibitory conditions mostly started out in pit mode, and shifted to trench mode without losing the presence of the SZ, thereby leading to resorption over longer periods and distances (Fig. 1A, Video 1; example 1 shows resorption in trench mode). In contrast, pit formation occurred as stationary resorption where the OC resorbs for several hours (average 12.5 ± 6.0 h) and then moves to another site following disassembly and reassembly of the SZ for a new resorption event (Fig. 1B, Video 1; control condition-example 2). Interestingly, changes in the resorption behaviour of OCs were similar with the ectosteric (T06) and active site-directed (ODN) CatK inhibitors. Both T06 and ODN further restricted extension/formation of trenches, which, therefore, remained as small trenches/pits (Fig. 1B,C; and Video 1). They induced a concentration-dependent reduction in the number of trenches, all the way down to their complete absence (Fig. 2A). Inversely, the number of OCs ceasing to make trenches increased (Fig. 2B). At roughly the IC_50_ concentrations of T06 (IC_50_ 245 ± 55 nM^19^) and ODN (IC_50_ 15.3 ± 8.8 nM^13^), OCs were still able to form short trenches but showed frequent stopping (Fig. 2B); they also engaged in forming multiple resorption events (Fig. 2F) indicating that approximately 50% CatK inhibition (IC_50_) still allowed bone resorption (50–60%), albeit reduced. However, at higher inhibitor concentrations (2 to 5 times the IC_50_ of tested inhibitors), OCs lost their ability to make trenches (Fig. 2A) and were limited to only making pits (Fig. 2E). Accordingly, the inhibition of CatK increased the number of multiple pits formed by the same OC (~ 75%) when compared to other OCs forming a single pit (~ 25%) (Fig. 2F,G). Under control conditions, most of the OCs initiated bone resorption in pit mode (Fig. 2D) and then the majority of them shifted into trench formation, whereas only a small fraction of OCs (~ 25%) were involved in direct trench formation (Fig. 2C). Parallel resorption experiments (end-point analyses) with live imaging for OCs from the same donors indicated that both T06 and ODN reduced the amount of eroded bone surface without affecting the number and viability of OCs after 72 h of culturing (Supplementary Fig. 1).

### Effect of CatK inhibition on the OC resorption speed

**Figure 3:**
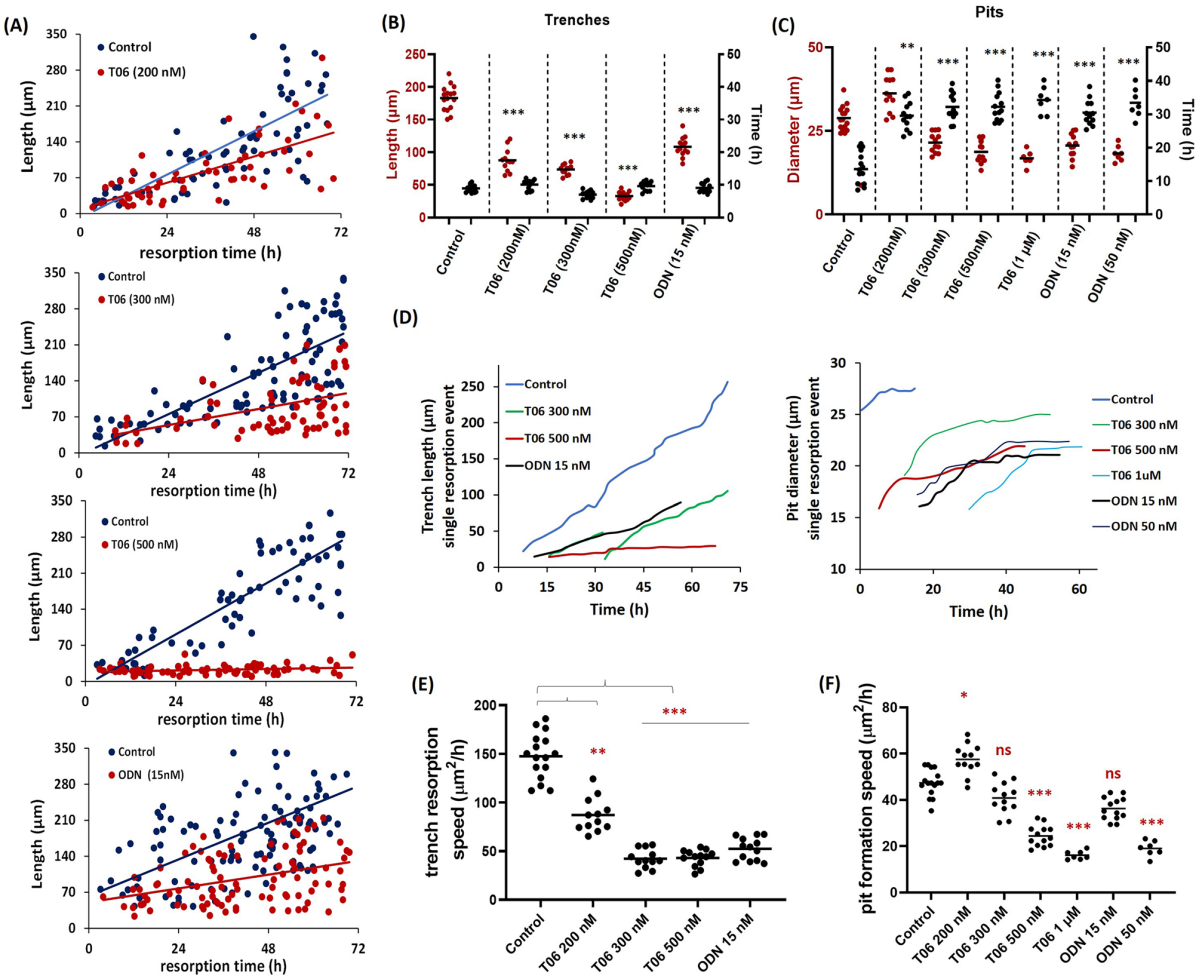
Relationship between duration and size of resorption events in the presence or absence of CatK inhibitors during a 72 h observation period. (**A**) Relationship between the length of trenches and the time of resorption in T06-treated (200 nM; n = 59, 300 nM; n = 97, 500 nM; n = 70), and ODN-treated (15 nM; n = 70) (numbers are per donor). OC cultures when compared with their untreated controls (n = 82 to 134) in one experiment. (**B**,**C**) Overview of the effects of CatK inhibitors on the duration (black dots) and diameter (red dots) of trench and pit resorption events in a series of experiments. Sample size: Control: n = 16 from 5 donors; 200 nM T06: n = 12 (3 donors); 300 nM T06: n = 12 (3 donors); 500 nM T06: n = 13 (4 donors); 1 µM T06: n = 7 [2 donors); 15 nM ODN: n = 12 (3 donors); 50 nM ODN: n = 7 (2 donors). For each donor, 3–5 replicate experiments on individual bone slices were analyzed for all conditions. We analyzed between 50 and 150 OCs per condition per experiment for each of the donors). Statistics: two-tailed Kruskal–Wallis’s test (ns, Not significant; **P = 0.008; ***P < 0.0001); Dunn’s multiple comparisons test (ns, Not significant; **P = 0.01; ***P < 0.001) compared to the untreated control. Significant reduction in the length/size of trenches was observed as shown in (**B**). However, the time span of OCs in pit formation mode after T06 and ODN treatment was significantly higher compared to untreated controls shown in (**C**). (**D**) Illustrative examples of the effect of CatK inhibitors on the progress of individual resorption events achieved by OCs in trench or pit resorption mode (length/diameter changes vs time). (**E**,**F**) Effect of CatK inhibitors on the resorption speed (µm^2^/h) of OCs in pit and trench mode in a series of experiments. Horizontal lines reflect the median. Statistics: two-tailed Kruskal–Wallis’s test, ns, not significant; *P = 0.034; **P = 0.0020; ***P < 0.0001; Dunn’s multiple comparisons test, ns, not significant; *P = 0.049; **P = 0.0045; ***P < 0.0001 was performed between groups to determine significance compared to the untreated control.

We consistently noted that resorbing OCs under control conditions were able to cover long distances in a short time-period and that inhibiting CatK resulted in smaller resorption events. Figure 3 A shows that the fitted line, reflecting distance (in µm) over time by individual OCs in trench-resorption mode, has a reduced slope in the presence of CatK inhibitors, i.e., a reduction in the speed of progression of trench-forming OCs over the bone surface. Quantification of the size (length and diameter in µm) and duration (h) of trench and pit resorption events in untreated and inhibitor-treated conditions is shown in Fig. 3B,C. The most pronounced effect of CatK inhibition on trench formation is the decrease in their size (Fig. 3B), and the strongest effect of CatK inhibition on pit formation is the increase in duration (Fig. 3C). These effects lead to a significant reduction in the speed of resorption (µm^2^/h) of both trench and pit formation (Fig. 3E,F). Analysis of the time course of individual pit and trench formation events (Fig. 3D) showed that the increase in length of formed trenches is higher than that of the increase in the diameter of formed pits; it also indicated that the increase remains constant in the case of trench formation, but decreases for pit formation during the observation period of 72 h. Furthermore, it was also observed that CatK inhibition reduces these size parameters for trenches but has little to no effect on pit sizes (Fig. 3D). In contrast, the pit formation speed for T06 at approximately the IC_50_ concentration (200 nM) is slightly increased compared to controls, despite spending a longer duration at the excavation site (Fig. 3F). We observed that OCs were involved in generating larger and irregular pits in the presence of 200 nM T06, which also resulted in an increased pit formation speed (Fig. 3C,F; Supplementary Fig. S2, Video S1). Furthermore, we tracked individual OCs for their subsequent activity and differentiated the effect of CatK inhibitors on the resorption speed of OCs involved in either generating a single pit, multiple pits, or pits switching into trenches. The resorption speed (µm^2^/h) of OCs generating pits that transformed into trenches is significantly higher than those forming individual pits or multiple pits, and a further reduction in resorption speed was observed with increased CatK inhibition (as shown in Supplementary Fig. S3, Video S2).

### CatK inhibitors at high concentrations induce an atypical resorption behaviour in OCs

**Figure 4:**
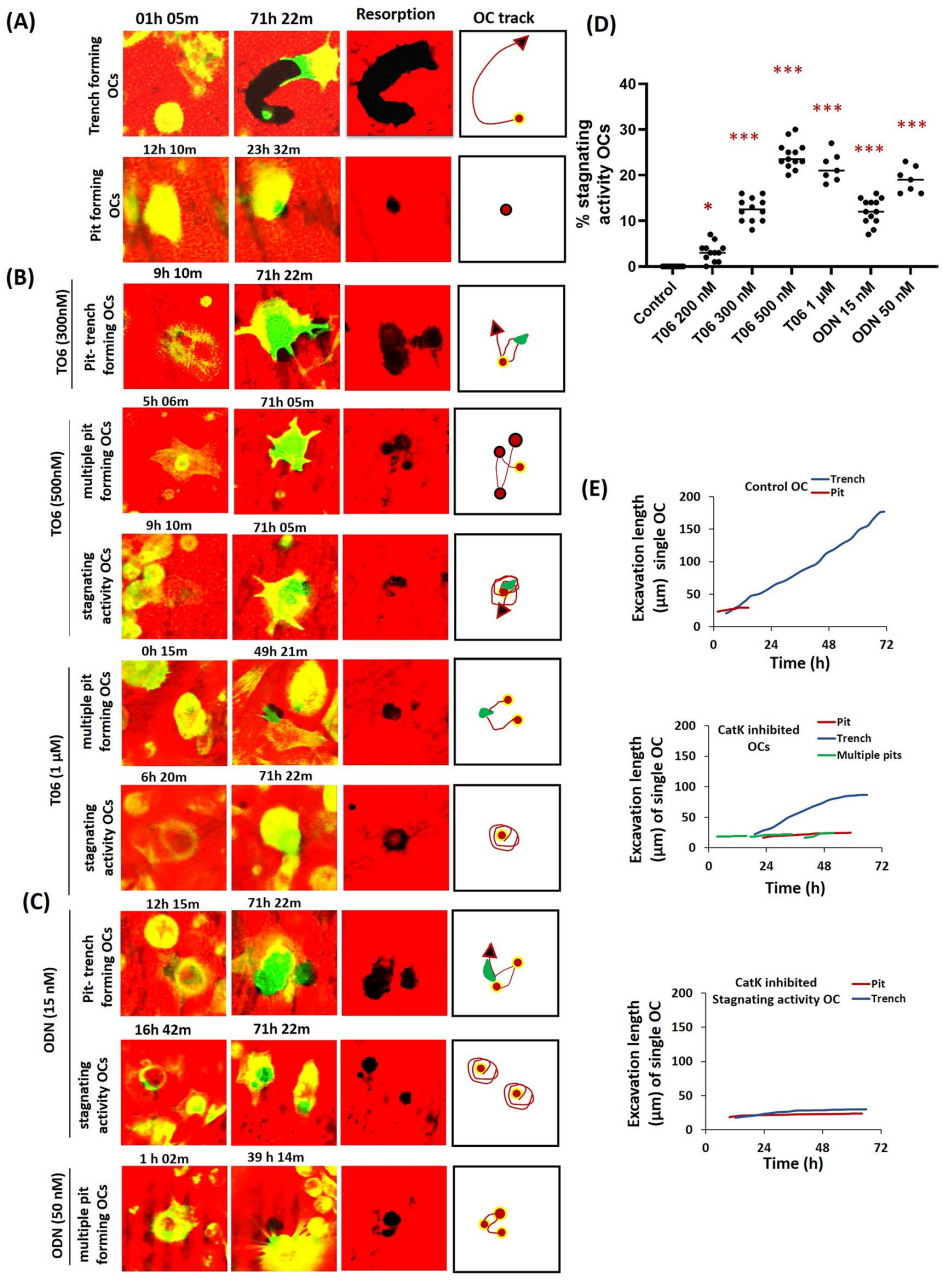
Induction of atypical bone resorption behaviour of OCs by CatK inhibitors at high concentrations. (**A**) Time-lapse images showing the typical behaviour of OCs in trench (upper panel, Video 2) and pit (lower panel, Video 2) resorption mode during a 72 h observation period. (**B**,**C**) CatK inshibition by the inhibitors (300 nM T06 [Video 2], 500 nM T06, 1 µM T06 [Video 3], 15 nM ODN, and 50 nM ODN [Video 4]) induces two prominent behaviors: multiple migration-resorption episodes resulting in adjacent pits and an atypical behavior characterized by slower/decelerating erosion and erratic displacements at the same excavation, suggesting an almost stagnating activity of OCs. OC displacement during the formation of resorption cavities is shown in the OC track column. Red dots indicate the initiation of resorption events, red arrows are the further direction of OC displacement, and green areas are the small trenches generated by the same OCs during their erratic displacement. (**D**) The frequency of OCs in “stagnating activation state” in untreated and inhibitor treated conditions. OCs in the stagnating activation state are mostly induced at high inhibitor concentrations, but already start appearing at lower concentrations. Horizontal lines reflect the median. Sample size: control: n = 16 from 5 donors; 200 nM T06: n = 12 (3 donors); 300 nM T06: n = 12 (3 donors); 500 nM T06: n = 13 (4 donors); 1 µM T06: n = 7 (2 donors); 15 nM ODN: n = 12 (3 donors); 50 nM ODN: n = 7 (2 donors). For each donor, 3–5 replicate experiments on individual bone slices were analyzed for all conditions. We analyzed between 50 and 150 OCs per condition per experiment for each of the donor). Statistics: two-tailed Kruskal–Wallis’s test (ns: not significant, *P < 0.05; ***P < 0.0001); Dunn’s multiple comparisons test (ns: not significant, *P < 0.05; ***P < 0.001) were performed between groups to determine significance compared to the untreated control. (**E**) The progression of single resorption events achieved by OCs in typical state (upper graph), inhibited state (middle graph: 300 nM T06), and stagnating activation state (lower graph: 500 nM T06). The x-axis is plotted from the beginning until the end of the time lapse observation. Single OC activity was tracked from the initiation of actin ring formation until the end of resorption. Most of the OCs initiate pit formation (~ 10–15 µm) and the subsequent change in the size of these excavations by typical and stagnating OCs is shown by these graphs. Note the atypical slow resorption speed of trench formation by OCs in stagnating activation state, which is in contrast to the constant resorption speed in typical trench resorption events.

Our data suggest that CatK inhibitors affect the currently known resorption modes; i.e., the relative proportion of pit-, multiple pit-, and trench-forming OCs, as well as the resorption speed and excavation size in each of these resorption modes. However, our time-lapse approach also allowed for the recognition of a previously unreported bone resorptive behaviour, characterized by (1) slow rhodamine removal, which decelerates with time in trench mode and (2) by persistent engagement of the OC at the same excavation, moving erratically and orienting its SZ/ruffeled border in multiple successive directions. This behaviour suggests a stagnating activity state, which involves both pit-forming OCs (circular movements) and trench-forming (back and forth movements) OCs. OCs in this stagnating activity state are compared to typical pit- (single/multiple) and trench-forming OCs in Fig. 4A–C, and in a series of videos: Untreated OC (Video 2), 300 nM T06 (Video 2), 500 nM T06, and 1 µM T06 (Video 3) as well as 15 nM ODN and 50 nM ODN (Video 4). These figures and videos show that CatK inhibition may induce erratic movements within the same excavation site. OCs in a stagnating activity state are not seen in control conditions, but start to appear above 200 nM T06 and 15 nM ODN concentrations (Fig. 4D). At 500 nM T06, they represent ~ 25% of the resorbing OCs. This is different from CatK inhibition inducing repeated resorption-migration cycles resulting in multiple pits. Figure 4E highlights that the resorption events achieved by OCs in stagnating activity states progress slowly (excavation length), and that the slow speed of trench enlargement is itself decreasing. This is in contrast to the constant elongation speed in typical trench resorption events in the absence of inhibitors (Fig. 4E). Simultaneous, consecutive images of OCs involved in forming excavations in untreated, 300 nM T06, 500 nM T06 (Video 3), 1 µM T06 (Video 3), and 50 nM ODN conditions (Video 4) are shown in Supplementary Fig. S4.

### Localization of active CatK in OCs and mode of action of ectosteric and active site-directed CatK inhibitors during bone resorption

**Figure 5:**
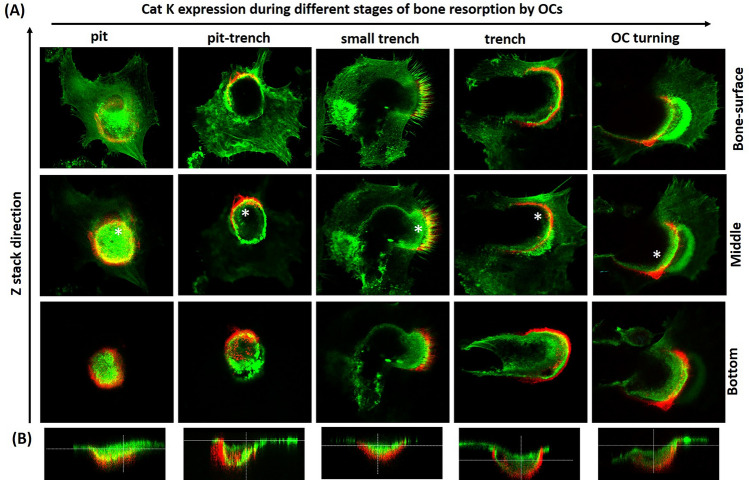
(**A**) Distribution of active CatK in OCs during different stages of bone resorption. CatK (Red: CatK, Green: Actin, Yellow: overlapped) can be localized at the ruffled border of OCs (green; actin). (**B**) A cross-section (xz) through the length axis of the OC involved in the generation of pits and trench. XZ sections in the direction of OCs resorption activity are highlighted with a white star (*) in the middle stack of each resorption stage.

Our observations highlight the existence of distinct OC resorption phenotypes, which reflect distinct spatial arrangements of the resorption machinery. This is especially obvious when observing OCs that start an excavation as a pit and then enlarge it by forming a trench (as shown in Fig. 5A, top arrow). Using a fluorescently-labeled peptidyl acyloxy ketone inhibitor (GB123) as a protease activity-based probe and z-stack confocal imaging, we localized the cathepsin activity during the successive stages of bone resorption events progressing from pit to trench. GB123 reacts the active site of cysteine cathepsins^37,38^. As CatK is the most abundant cathepsin in OCs^8,9^, staining predominantly represents this protease. This is also corroborated by the finding that the selective active site-directed CatK inhibitor, ODN, completely abolished CatK labelling by GB123 above 3 times higher IC_50_ concentrations (50 nM), whereas even at a 5 times higher IC_50_ concentration of the ectosteric CatK inhibitor, T06 (1 µM), no loss of the active site activity of CatK was observed (Supplementary Fig. S5). At all stages, most of the active CatK was positioned at the invasive front of the ruffled border (Fig. 5A top view and B side view). When the OC makes a pit, CatK activity shows circular distribution corresponding with the inner edge of the SZ at the bone surface level and with the resorption compartment all around the ruffled border, consistently from top to bottom. When the OC shifts from pit to trench resorption mode, the SZ orients the ruffled border against the side of the cavity wall to be resorbed while the leading edge of the SZ is kept on the bone surface. CatK activity is then polarized at the inner side of the leading edge of the SZ, spreading in a crescent-like shape just where cavitation starts. The highest activity signal remains at the bottom of the cavity, along the wall that is being invaded by the ruffled border. Any change in the direction of the resorbing OC is associated with a corresponding change in position of the active CatK (Fig. 5 right panel indicating an OC turn, as visualized by the CatK location). Thus, the position of active CatK indicates the direction of resorption.

### Fusion of OCs during bone resorption and its effect on resorption speed

**Figure 6:**
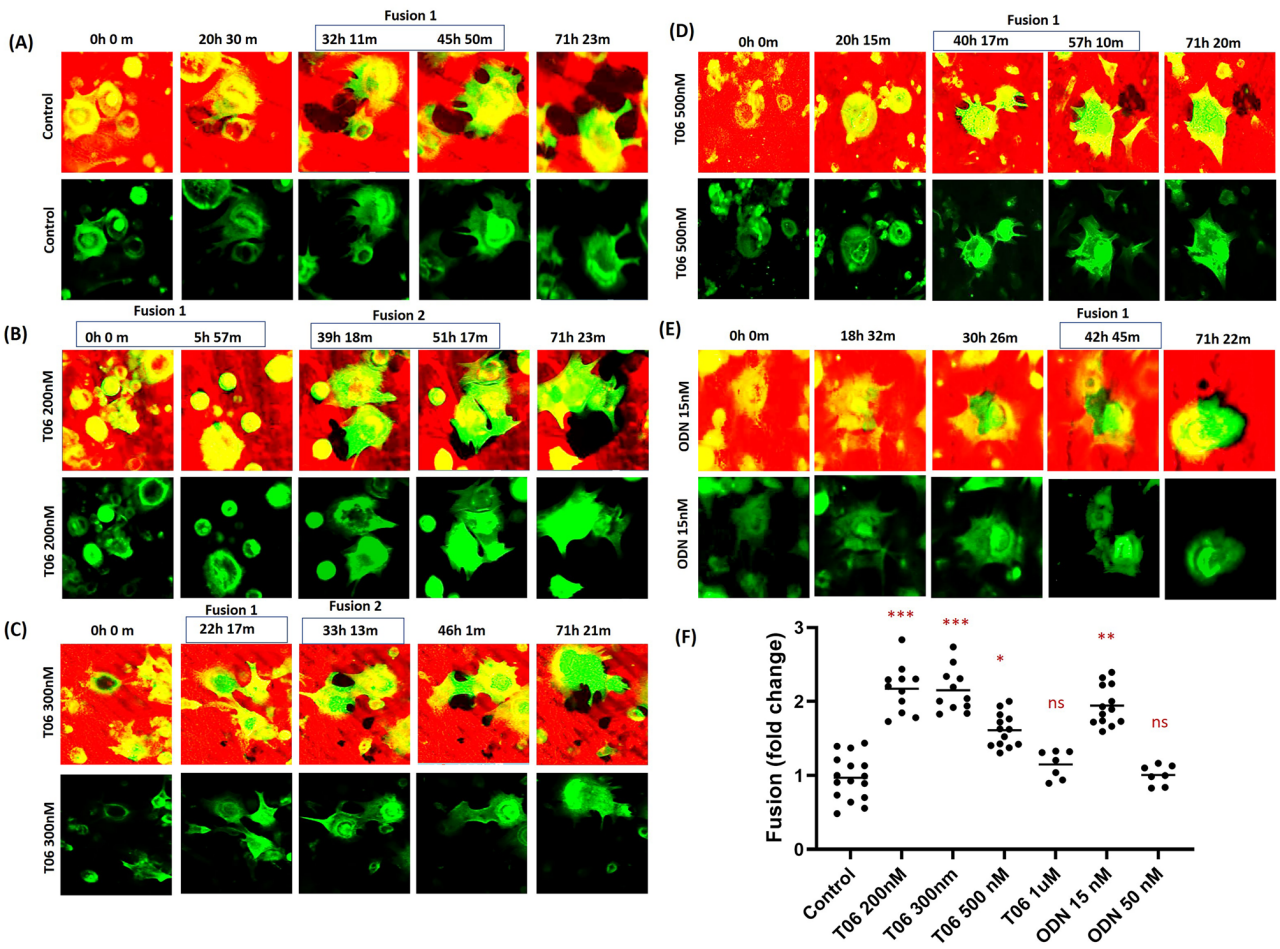
Fusion events of OCs during bone resorption activity. Time-lapse images taken from the videos of the (A) untreated control (Video 5), (**B**) T06 200 nM (Video 5), (**C**) T06 300 nM (video 5), (**D**) T06 500 nM (Video 5), and (**E**) ODN 15 nM (Video 5) treated cultures. Fusion of OCs increases at IC_50_ and lower concentrations of both CatK inhibitors. Pit- and trench-forming OCs were stained for actin by using phalloidin (green), and the bone surface was stained with rhodamine (red). Resorption events generated by OCs are shown in the upper panel and OC movement and fusion can be clearly seen in the lower panel in each condition. Time-lapse videos show that most of the OCs make long trenches in the absence of CatK inhibitors. However, in the presence of CatK inhibitors smaller resorption events and increased cellular motility around the resorption cavity resulting in increased cell fusion to compensate for the decreased resorption activity are observed. OCs show increased fusion events at T06 200 nM, T06 300 nM, and ODN 15 nM concentrations, which diminishes at higher inhibitor concentrations. (**F**) Quantification of fusion events in untreated and inhibitor-treated conditions (control: n = 16 from 5 donors; 200 nM T06: n = 12 (3 donors); 300 nM T06: n = 12 (3 donors); 500 nM T06: n = 13 (4 donors); 1 µM T06: n = 7 (2 donors); 15 nM ODN: n = 12 (3 donors); 50 nM ODN: n = 7 (2 donors). For each donor, 3–5 replicate experiments on individual bone slices were analyzed for all conditions. We analyzed 50–150 OCs per condition per experiment for each of the donor). Statistics: two-tailed Kruskal–Wallis test (*P = 0.02, **P = 0.005, ***P < 0.0001; Dunn’s multiple comparisons test (*P = 0.05; **P = 0.008; ***P < 0.0001) was performed between group to determine significances compared to the untreated control.

We noticed that fusion events during bone resorption occur and enhance resorption speed. Fusion happens in control conditions but becomes more frequent in the presence of low concentrations of CatK inhibitors (Fig. 6A–E; control, 200 nM T06, 300 nM T06, 500 nM T06, 15 nM ODN (Video 5). Quantifications showed an approximate twofold increase in the fusion events after CatK inhibition at the corresponding IC_50_ concentrations of both inhibitors (200 or 300 nM T06 and 15 nM ODN) when compared to control conditions (Fig. 6F). At higher inhibitor concentrations, where bone resorption is almost completely inhibited, the fusion rate is reduced and is similar to the fusion rate of uninhibited OCs (Fig. 6F).

**Figure 7:**
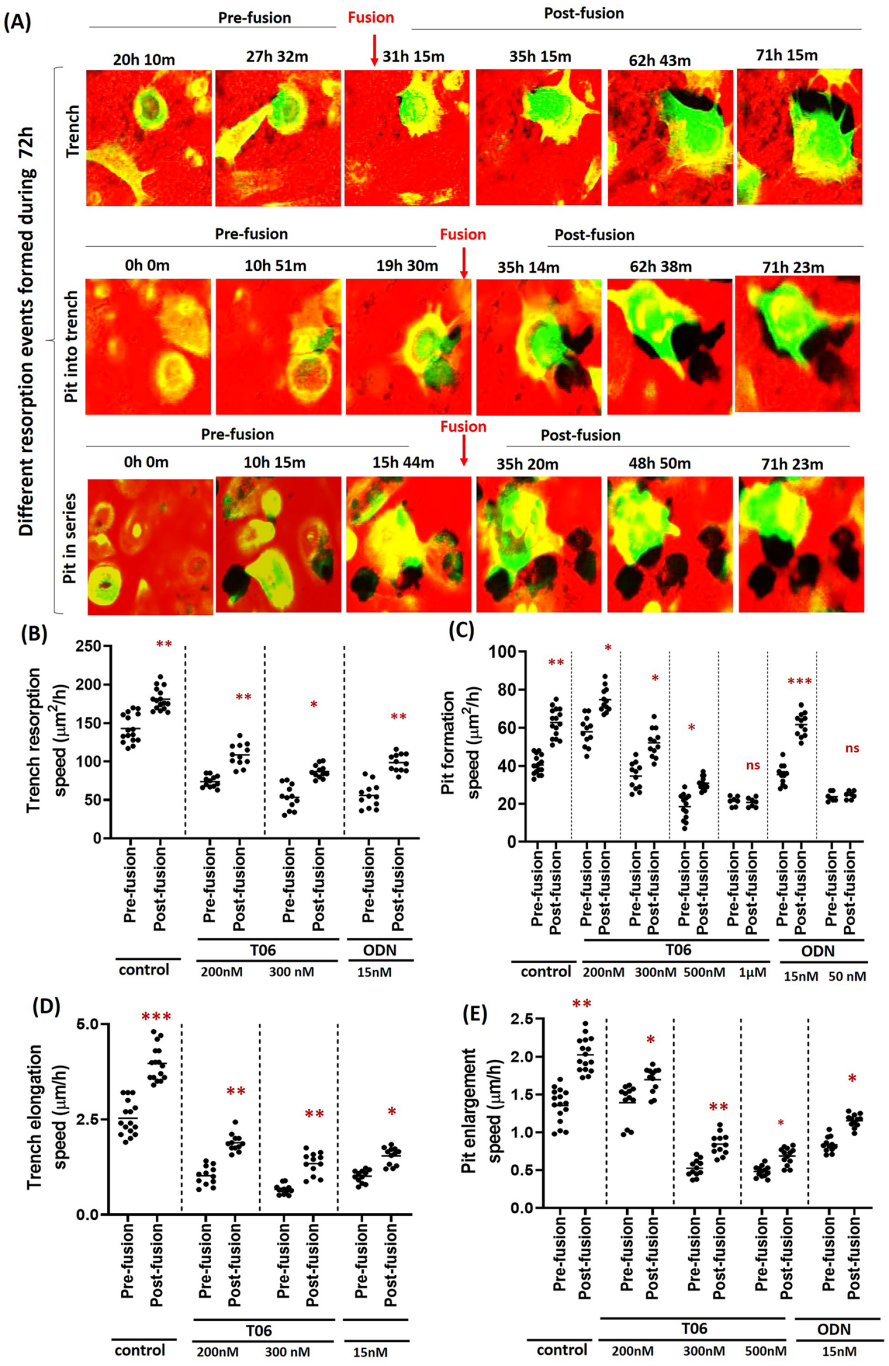
Effect of fusion on OC movement during bone resorption. (**A**) Time-lapse images showing the fusion of OCs while making trenches, pits transforming into trenches, and during multiple pit formation (Video 6). (**B**,**C**) Graphs displaying the increase in the trench- and pit-associated resorption speed of OCs immediately after fusion in the absence and presence of CatK inhibitors. (**D**,**E**) Graphs showing the migration speed of OCs in the absence and presence of T06 and ODN confirm that increases in the resorption speed of OCs via fusion are not because the OCs increased in size due to fusion, but due to their faster movement as a result of fusion. Fusion events are reduced at higher inhibitor concentrations. Data presented are from: control: n = 16 from 5 donors; 200 nM T06: n = 12 (3 donors); 300 nM T06: n = 12 (3 donors); 500 nM T06: n = 13 (4 donors); 1 µM T06: n = 7 (2 donors); 15 nM ODN: n = 12 (3 donors); 50 nM ODN: n = 7 (2 donors). For each donor, 3–5 replicate experiments on individual bone slices were analyzed for all conditions. We analyzed 50–150 OCs per condition per experiment for each of the donors. Statistics: two-tailed Mann–Whitney test (ns, Not significant; *P < 0.05; **P < 0.01; ***P < 0.001).

Furthermore, fusion occurs irrespective of whether the OC is engaged in trench formation, transition from pit to trench mode, or transition from pit-to-pit mode (Fig. 7A and Video 6). A remarkable observation is that OC fusion during bone resorption significantly increases the resorption speed of OCs in untreated and inhibitor-treated conditions up to their IC_50_ range. This is shown by quantification of the pre- and post-fusion resorption speed of the OCs over the bone surface in different conditions (Fig. 7B,C). Figure 7D,E shows that increased resorption speed in trench and pit mode directly relates to the acceleration of movement of OCs over the bone surface, and does not simply result from wider resorption tracks due to the larger size of the OC after fusion (Fig. 7A). At high inhibitor concentrations, where CatK is mostly inhibited and the resorption activity is limited to a reduced pit mode, the fusion rate becomes similar to non-inhibited OCs (Figs. 6F and 7C).

## Discussion

Inhibition of bone resorption by cysteine protease inhibitors was classically ascribed to the decreased degradation of triple-helical collagen, which then accumulates and prevents further invasion of the ruffled border into the bone matrix^39^. Upon the discovery of CatK in the mid-1990s, it became clear that CatK with its unique collagenase activity and its predominant expression in OCs, is the key protease in bone resorption^8–10,40^. When the original mechanistic model was conceived, resorption was believed to be “stationary” leading to resorption “pits”. At present, there is increasing awareness that a “mobile” resorption mode, driven by moving OCs and leading to resorption trenches, greatly contributes to overall resorption^7,27,41–45^. However, the “mobile” resorption mode, which appears especially sensitive to CatK inhibitors, is missed when OC cultures are assessed only at the end of the experiment^13,19–21,23^. The present study is focused on real-time imaging which is highly suitable to evaluate the effect of CatK inhibitors on the behaviour, mobility, and resorptive activity of OCs during the entire resorption process.

Our videos highlight that CatK inhibition reduces OC mobility but does not prevent the formation of pits. OCs subjected to CatK inhibition remain connected with the pit for 2–3 times longer periods, compared to control conditions. These observations are in accordance with the view that CatK inhibition results in an accumulation of demineralized collagen, which acts as a barrier preventing the penetration of the ruffled border into the bone matrix^22^. Our observations also confirm the sequence of events reported to occur after pit formation^22^: either the OCs lose their annular SZ (which also reflects the loss of their ruffled border and resorption compartment), migrate away, and may start making a new pit close-by, or alternatively, the OCs change their SZ from annular to crescent-like (which reflects continued lateral resorption in trench mode). Interestingly, the frequency of OCs switching from an annular to a crescent-like SZ and subsequently starting progressive trench formation is greatly reduced during CatK inhibition, while OCs showing multiple pit formations are greatly increased. Thus, these concurrent changes explain well why CatK inhibition results in decreased trench surfaces and concomitantly increased pit surfaces, as shown earlier by measuring the relative prevalence of pits and trenches at the end of the cultures^13,20–23,42^.

An intriguing question raised by the present observations is how CatK activity affects parameters like (1) the orientation of the ruffled border/SZ, which determines whether a pit or trench is formed; (2) the distance travelled by the ruffled border over the bone surface; and (3) the speed of this travel. It appears likely that the collagen degradation activity of CatK is responsible, since T06, which specifically inhibits the collagenolytic activity of CatK^14,19^, is shown here to affect all these parameters. The effect on bone resorption is extremely similar to that seen for ODN, which inhibits the entire proteolytic spectrum of CatK, and, thus the non-collagenolytic activities of CatK may not contribute significantly to the observed OC behaviour. Furthermore, the emerging model, describing how OC resorption pits are enlarged into trenches^24^, provides a scenario explaining how CatK collagenolysis governs the SZ/ruffled border dynamics. In brief, slowing down collagenolysis leads to the accumulation of demineralized collagen in the pit which may limit the displacement of the SZ within the pit. This could be due in part to the inhibition of the CatK-catalyzed exposure of type I collagen cryptic arginine-glycine-aspartate (RGD) domains involved in the attachment of the ruffled border to the matrix via the podosome-associated αvß3 integrins^44,46,47^. The displacement of parts of the SZ in the pit is mandatory to orient the ruffled border against the cavity wall and to initiate trench formation^24^. Similarly, continuous displacement of the SZ parallel to the bone surface depends on thorough collagen removal from the cavity wall and the exposure of new CatK-exposed RGD domains for the binding of the integrins located in the ruffled border during the progress of resorption. Thus, non-degraded demineralized collagen on the cavity wall will make trench elongation stop and result in shorter trenches. The need for thorough collagen removal from the cavity wall was supported earlier by SEM, spectroscopy, and collagen immunoreactivity^13,48^, and is further supported here by the polarization of active CatK at the front line of the cavitating activity. Of note, the results obtained with a fluorogenic inhibitor probe binding to the active site of CatK unambiguously inform on the position of active CatK in bone resorbing OCs as earlier reported by Zhuo et al.^23^.

The present time-lapse observations also revealed a previously unreported OC activity, which we called the “stagnating” activity state. It is only seen in response to CatK inhibition: OCs remain associated with the same excavation, showing the SZ moving erratically in multiple successive directions, with minimal resorption. This behaviour is in contrast to the situation where CatK inhibition induces OCs to make multiple adjacent pits. The mechanism determining these respective behaviours remains to be investigated.

Our video observations provided evidence that OCs fuse while resorbing, that the frequency of fusion events increases in the presence of moderate concentrations of CatK inhibitors, and that these fusions induce acceleration of trench and pit elongation and expansion, respectively. To the best of our knowledge, these results were never reported before, and they may give a mechanistic explanation for the larger OCs obtained with in vitro ODN treatment observed by Zhou et al.^23^. We speculate that such an enlargement of the OCs due to reactivation of cell fusion represents a feedback mechanism to compensate for impaired collagenolysis, as a relationship between the number of nuclei and CatK levels has been reported previously^29^. The molecular mechanism linking CatK activity and cell fusion remains to be investigated. One possible explanation could be that the accumulation of demineralized collagen contributes to their fusion since collagen/OC interactions promote fusion^34^ and OC fusion is impaired in the absence of the collagen receptor OSCAR^32^. Our findings may also give a mechanistic explanation for the long-standing observations that OCs in bones of patients undergoing treatment with bisphosphonates may appear large and more nucleated than those of controls and show signs of residual bone resorption^49–51^.

It is important to know that there is a great intrinsic variability in CatK levels amongst individuals, and that titration experiments showed that these differences can explain 68% of the variation in prevalence of trench forming OCs amongst studied individuals^20^. These variations may affect OC resorptive activity as shown in this study through the pharmacological manipulation of CatK activity levels. Furthermore, conditions like glucocorticoid and bisphosphonate treatment, menopause, and aging were found to be associated with both upregulation of CatK levels in the OCs and higher prevalence of trench formation^25,26,31^.

There were limitations to this study. While we acknowledge that an in vitro model system cannot include the complexity of in vivo experiments (discussed previously in^24^), we may argue that this reductionist model might be best suited to study the direct effects of CatK inhibitors on the resorptive activity of individual OCs. However, co-cultures of OCs and osteoblast lineage cells have shown that osteoblastic collagenases also influence the resorption mode of the OC^52^. Thus, when it comes to in vivo scenarios, where OCs are surrounded by osteoblast lineage cells, other collagenases should also be taken into account.

In conclusion, live imaging of the response of OCs to CatK inhibition demonstrates a key role of CatK collagenolysis for regulating the “navigation” of OC resorptive activity over the bone surface. CatK-catalyzed collagen degradation determines the direction, magnitude, and speed of resorption, thereby greatly influencing the aggressiveness of the OC resorptive activity. Furthermore, it was recognized for the first time that a partial reduction in the level of active CatK promotes OC fusion, which in turn induces acceleration of the resorption over the bone surface. The present observations point to CatK as an ideal target of anti-resorptives, since high levels of CatK render the OC particularly aggressive by specifically promoting enlargement of the resorption cavities and the resorption speed, which are precisely the failures/malfunctions of OCs in individuals during menopause and aging. Thus, CatK inhibitors antagonize exactly what renders bone resorption aggressive in these situations. Ectosteric CatK inhibitors, like the tanshinone T06, are promising candidates since they inhibit collagen degradation without inhibiting the activity of CatK on other substrates^19^, thereby potentially avoiding the side effects of classic active site-directed CatK inhibitors like Balicatib and ODN, which therefore failed in their Phase 2 and 3 clinical trials^53,54^.

## Materials and methods

### OC preparation

CD14^+^ monocytes were isolated from human blood of 5 different donors and differentiated into mature OCs as described previously^27,55^. Anonymized buffy coats from healthy blood donors were used in accordance with Danish legislation. All donors gave written informed consent for the use of surplus material from the donation. This procedure of using an anonymous donor with consent has been approved by the Danish Ministry of Health and the Regional Ethics Committee of Southern Denmark. In brief, the Ficoll-Plaque separation method (GE Healthcare, UK) was used, and cells in the interphase were collected, suspended in PBS containing in 2 mM EDTA, and purified using BD IMag™ Anti-Human CD14 magnetic particles (BD Biosciences, CA, USA) according to supplier instructions. CD14^+^ cells were seeded at a density of 5 × 10^6^ cells in T75 culture flasks supplied with α-Minimum Essential Medium (αMEM; Invitrogen) containing 10% fetal calf serum (FCS; Sigma-Aldrich) and 25 ng/ml human macrophage colony-stimulating factor (M-CSF; R&D Systems), then cultured at 37 °C for 2 days in 5% CO_2_ in a humidified incubator. Subsequently, the medium was renewed, supplemented with 25 ng/ml of both M-CSF and RANKL, and cultured for another 7 days (with medium renewal performed twice) to get mature OCs.

### Time-lapse recordings

Time-lapse recordings were done as described previously^27^. Cortical bovine bone slices (0.4 mm; BoneSlices.com, Jelling, Denmark) were labeled with rhodamine fluorescent dye (ThermoFisher) as previously described^27^. Mature OCs were detached with accutase (Biowest BW, France), harvested by centrifugation (500 g for 5 min), and resuspended in αMEM containing 10% FCS, 25 ng/ml M-CSF, and 25 ng/ml RANKL. For control and lower inhibitor concentrations cells from 3 to 5 donors were used and for the highest inhibitor concentrations (T06 1 µM and ODN 50 nM) 2 donors were used. For each condition and for each donor 3–5 bone slices were used and cells were seeded at a density of 100,000 cells per bone slice in a 96-well plate. In order to label F-actin in living OCs, 100 nM SiR-actin (excitation at 652 nm; emission at 674 nm) and 10 μM verapamil (both supplied by Spirochrome, Stein am Rhein, Switzerland) were added and incubated for 5 h at 37 °C in 5% CO_2_ in a humidified chamber. Inhibitors (ODN and tanshinone IIA sulfonate (T06), which block CatK activity, were added to the plates. Subsequently, bone slices were transferred to Nunc Lab-Tek™ II Chambered Coverglass (ThermoFisher Scientific) wells in medium containing M-CSF, RANKL, SiRactin, and verapamil (with or without inhibitors) as described above. Time-lapse images were made using an Olympus Fluoview FV10i microscope (Olympus Corporation, Shinjuku, Tokyo, Japan) at 5% CO_2_ and 37 °C, with a 10 × objective lens with a confocal aperture of 2.0 corresponding to a z-plane depth of 20.2 μm. The initial focus was set to the bone surface. Recordings were made for a period of 72 h taking images every 7 or 21 min (at least 3 recording areas per bone slice). Neither SiR-actin nor verapamil affected the extent of resorption. We analyzed between 50 and 150 OCs per condition on 3–5 bone slices per donor-derived OCs.

### Analyses of time-lapse recordings for bone resorption

Analyses were performed as previously described^27^. An Olympus Fluoview 4.2 Viewer (Olympus Corporation) was used to optimize the intensities of each image series, and channels were further exported into “.avi” format. These videos were analyzed with ImageJ software (NIH, USA) for resorption length and area measurements in pixels. Subsequently, using information from the Olympus Fluoview 4.2 Viewer data manager, these were converted into µm. The beginning and end of each resorption event was estimated by counting the frames. Since there were either 7.0 or 21.7 min between each frame, these could be converted into hours and minutes. We previously observed that rhodamine labeling (of collagen) of the bone surface penetrated 5.7 ± 0.8 μm into the bone^27^, so complete removal of red surface staining by OCs meant that the collagenolysis had reached depths of ~ 5 μm or more. OCs that displayed a round actin ring and remained stationary were defined as pit-forming OCs. In contrast, OCs that initiate resorption as a pit by forming a circular stationary actin ring and shifting into lateral resorption—as determined by formation of a crescent-like shaped actin ring at one side of the pit—were defined as trench-forming OCs. Resorption during trench mode expanded in only one direction through the uptake of collagen by OCs.

In general, OCs initiate bone resorption by forming a circular F-actin ring. At the end of the resorption process OCs are ready to migrate away from the resorption area, which is detected by the disappearance of the actin ring followed by movement. Pits were defined as single or multiple stationary resorption events, which could be generated by a single or multiple OCs. Trenches start as a pit by OCs forming a circular actin ring, with the subsequent expansion of the pit into trenches through the actin ring being engaged into a forward moving direction (taking the form of a crescent-shaped actin ring). The area of individual resorption events (pits or trenches) was determined manually by tracing the edges of the cavities appearing as “black” due to removal of rhodamine-stained collagen. The areas of a trench comprise the full area of the trench minus the initial pit area. Length measurements for resorption cavities were done by manually drawing a line along the center of the resorption trail. “Stopping” was defined as the percentage of OCs discontinuing their trench mode, where the following factors were considered when calculating OCs stopping: (1) OCs switching from pit to trench mode and vice versa without generating long trenches, (2) OCs disassembling their SZ, and (3) a lack of rhodamine removal by OCs within 8 consecutive frames (21.7 min each frame for total of 2.9 h).

Emphasis was placed on observing the resorption pattern, size/area, and number of resorption events formed by OCs in the presence or absence of CatK inhibitors. OCs were monitored for the entire 72 h of recording, and their migration and behavior in response to different inhibitor concentrations were recorded. Effect of T06 and ODN on OCs was determined in parallel experiments using cell viability assay. After 72 h of incubation cells were fixed in 4% formaldehyde and subsequently stained for TRAcP activity using a leukocyte acid phosphatase (TRAP) kit (Sigma-Aldrich) and OCs (2 nuclei or more) were counted in all conditions. Bone slices were incubated in water and cleaned with a cotton stick and stained with toluidine blue to determine the eroded surface using a Nikon Eclipse Ci microscope.

### Analyses of OC fusion and its impact on bone resorption via time-lapse recordings

The track of each OC involved in bone resorption (in the presence or absence of CatK inhibitors) was analyzed in order to count the number of fusion events occurring during pit mode, transition from pit to trench mode, and during trench mode. By tracking each OC with their respective resorption cavity, we determined how often these OCs fuse in the presence or absence of CatK inhibitors. To gouge the impact of cell–cell fusion on bone resorption, we determined the length and area of resorption cavities (as mentioned previously) considering the dimension of the cavities pre- and post-fusion. Furthermore, we calculated the distance covered by OCs pre- and post-fusion per hour giving an estimate of changes in bone resorption speed preceding cell–cell fusion.

### Confocal microscopy

OCs at a density of 100,000 cells were seeded on 0.2 mm thick bone slices (BoneSlices.com, Jelling, Denmark) and incubated in αMEM containing 10% FCS, 25 ng/ml M-CSF, and 25 ng/ml RANKL for 72 h in the presence or absence of CatK inhibitors at different concentrations. At the end of the experiment, an activity-based probe for cathepsin, GB123 (provided from Galia Blum^37,38^), with a Cy5 tag (Ex/Em: 633/665 nm) as added to the culture media and incubated at 37 °C for 15 min. Subsequently, OCs were washed in PBS, fixed in 4% paraformaldehyde and 2% sucrose for 15 min at room temperature, washed, blocked/permeabilized (PBS, 0.5% BSA, 0.05% saponin), and incubated with phalloidin AF488 (A12379, Invitrogen). Bone slices were mounted on glass slides with a coverslip using ProLong Gold containing DAPI (Invitrogen). Confocal visualization of active CatK, together with F-actin, in fixed OCs on bone slices was performed (resolution xy-plane: 0.25 µm/pixel) using a Leica SP5 X Laser Scanning Confocal Microscope and HCX PL APO 63 × oil objective lens. Images were processed using LAS X Life Science Microscope and ImageJ software.

### Statistical analyses

Three to five independent experiments (cells generated from different human donors in each experiment) were recorded for 72 h in the presence or absence of ectosteric (T06) or active site-directed (ODN) CatK inhibitors. For each donor 3–5 replicate experiments on individual bone slices were analyzed for all conditions. All statistics shown in the graphs were performed using GraphPad Prism (version 9) software (GraphPad Software, San Diego, CA, USA) and SigmaPlot software (SPSS Inc.). The level of significance was defined as *P* < 0.05. Data sets were analyzed for normal distribution following a Gaussian distribution; the D’Agostino-Pearson omnibus test and statistical tests were adapted accordingly. Data are presented as medians. Two-tailed Kruskal–Wallis test, the Dunn’s multiple comparisons test, and the Mann–Whitney test were used in these analyses. More details on the employed statistics are given in the figure legends.

### Declaration of transparency and scientific rigour

This declaration acknowledges that this paper adheres to the principles for transparent reporting and scientific rigour of preclinical research as stated in the Scientific Reports guidelines for design & analysis, immunostaining, and as recommended by funding agencies, publishers and other organizations engaged with supporting research.

### Supplementary Information

Supplementary Video S1.

Supplementary Video S2.

Supplementary Information.

Supplementary Video 1.

Supplementary Video 2.

Supplementary Video 3.

Supplementary Video 4.

Supplementary Video 5.

Supplementary Video 6.

Supplementary Video Legends.

## Data Availability

Data are available in the article and supplementary file. Any data supporting the present study are available from the corresponding authors upon rational request.

## Abbreviations

OC: Osteoclast
CatK: Cathepsin K
SZ: Sealing zone
ODN: Odanacatib
nM: Nanomolar
µM: Micromolar
M-CSF: Macrophage colony-stimulating factor
RANKL: Receptor activator of nuclear factor kappa-Β ligand
