# Supplementary material for: Real-time analysis of osteoclast resorption and fusion dynamics in response to bone resorption inhibitors
Source: Sci Rep. 2024 Mar 28;14:7358. doi: 10.1038/s41598-024-57526-9 (PMC10978898; doi:10.1038/s41598-024-57526-9)
Supplement: Supplementary file 3 — Supplementary Information. [file 41598_2024_57526_MOESM3_ESM.docx]

**Supplemental material**

Fig. S1 displayed the effect of T06 and ODN on the reduction of eroded bone surface (%) without affecting TRAcP positive OC numbers and their cell viability at different concentrations. Fig. S2 (Video S1) displayed that with T06 (200 nM) inhibition, larger and irregular pits were generated by OCs which contributed to increased pit-formation speed (supporting data for Fig. 3). Fig. S3 (Video S2) displayed that the resorption speed of OCs in pit mode is related to their subsequent resorptive activity; OCs that generate the pits that transformed into trenches showed the highest resorption speed. Fig. S4 shows the consecutive images of OCs involved in forming resorption excavations during 72 h in typical and slow resorbing mode (stagnating activity) after CatK inhibition by T06 and ODN (supporting data for Fig. 4 and relevant videos). Fig. S5 displayed the CatK activity in resorbing OCs using an activity-based inhibitor probe (GB123) in the presence or absence of T06 and ODN at different concentrations (supplemental data for Figs. 5).


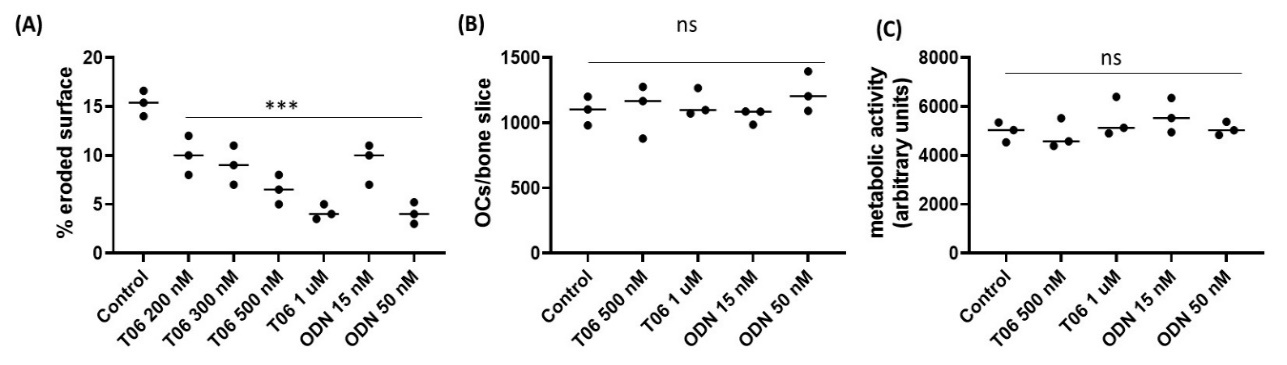


**Supplementary Fig. S1**: **Quantification of (A) % bone eroded surface, (B)TRAcP+ OCs, and (C) metabolic activity of OCs under different conditions in presence and absence of inhibitors (T06 and ODN at different concentrations).**


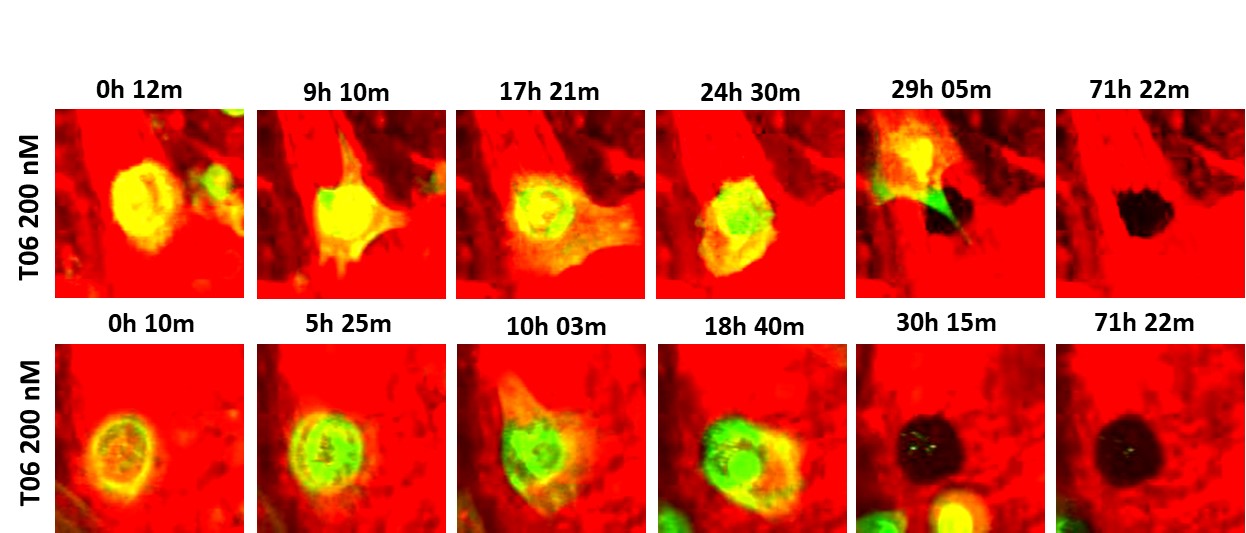


**Supplementary Fig. S2**: **Time-lapse images were taken from videos (Video S1) for a single pit with T06 (200 nM) inhibition displaying larger and irregular pits generated by OCs, which contributed to increased pit-formation speed.**

**Video S1:** Time-lapse videos of OCs generating irregular and larger pits with T06 (200 nM) inhibition shown in two examples. OCs are shown in green and bone surface (collagen) in red. Resorption by OCs over the bone surface is shown by black imprints (trenches and pits).


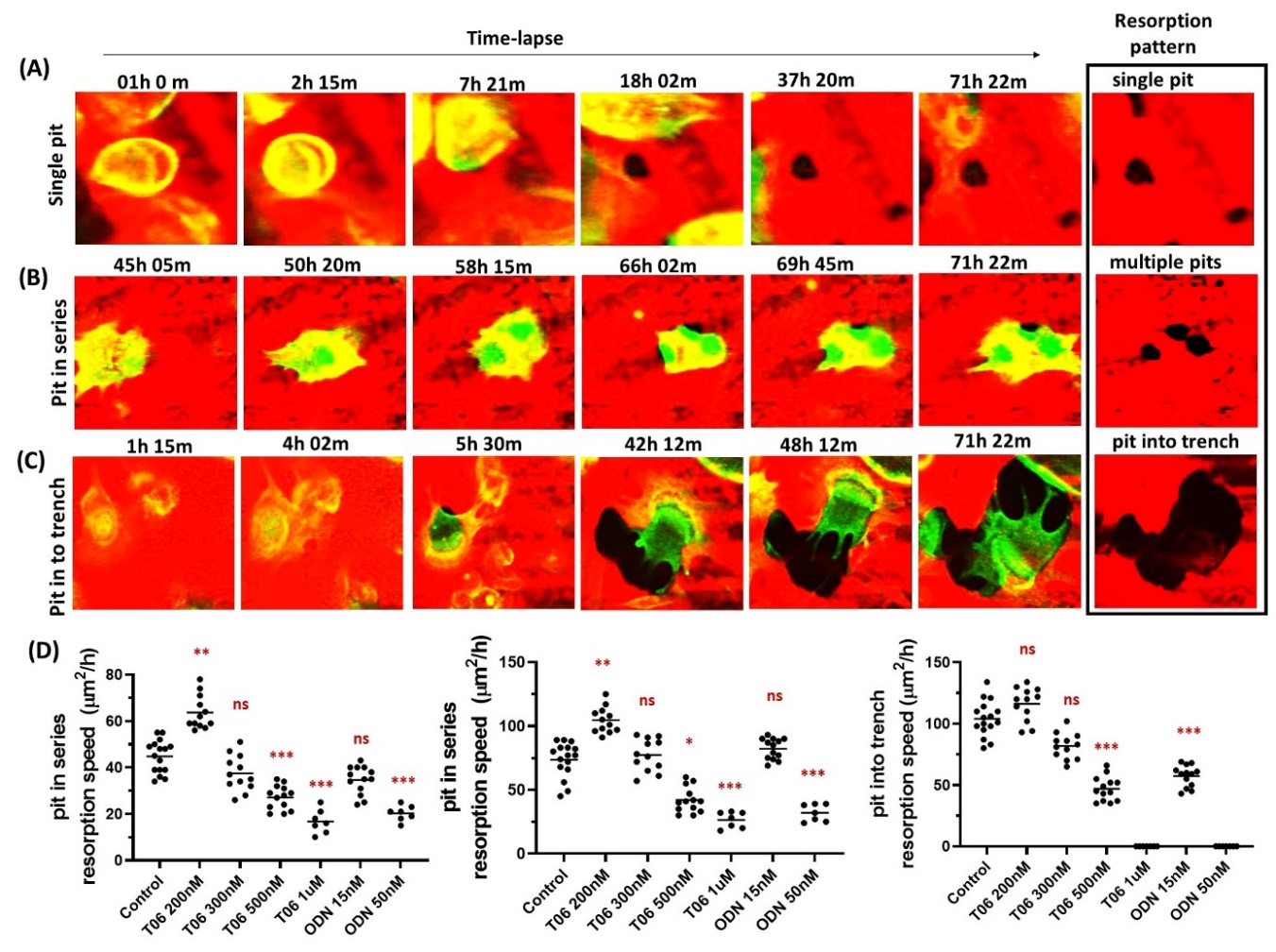


**Supplementary Fig. S3**: **Resorption speed of OCs in pit mode is related to their subsequent resorptive activity.** (A-C) Time-lapse images were taken from videos for a single pit, a pit in series, and a pit transformed into trench mode (Video S2) in untreated condition. (D) Resorption speed for pit formation in the presence or absence of T06 and ODN. OC activity was categorized according to how many single pits, pits in series, and pits into trenches were made. Sample size: control: n=16 from 5 donors; 200 nM T06: n=12 (3 donors); 300 nM T06: n=12 (3 donors); 500 nM T06: n=13 (4 donors); 1 µM T06: n=7 (2 donors); 15 nM ODN: n=12 (3 donors); 50 nM ODN: n=7 (2 donors). For each donor 3-5 replicate experiments on individual bone slices were analyzed for all conditions. We analyzed 50-150 OCs per condition per experiment for each of the donors. The median speeds obtained in each experiment are shown as dots. Statistics: A two-tailed Kruskal-Wallis test (ns: not significant; *P < 0.05; **P < 0.01; ***P < 0.0001) was compared to the untreated control.

**Video S2:** Time-lapse videos of OCs generating single pit, pit in series, and pit transformed into trench mode. OCs are shown in green and bone surface (collagen) in red. Resorption by OCs over the bone surface is shown by black imprints (trenches and pits).


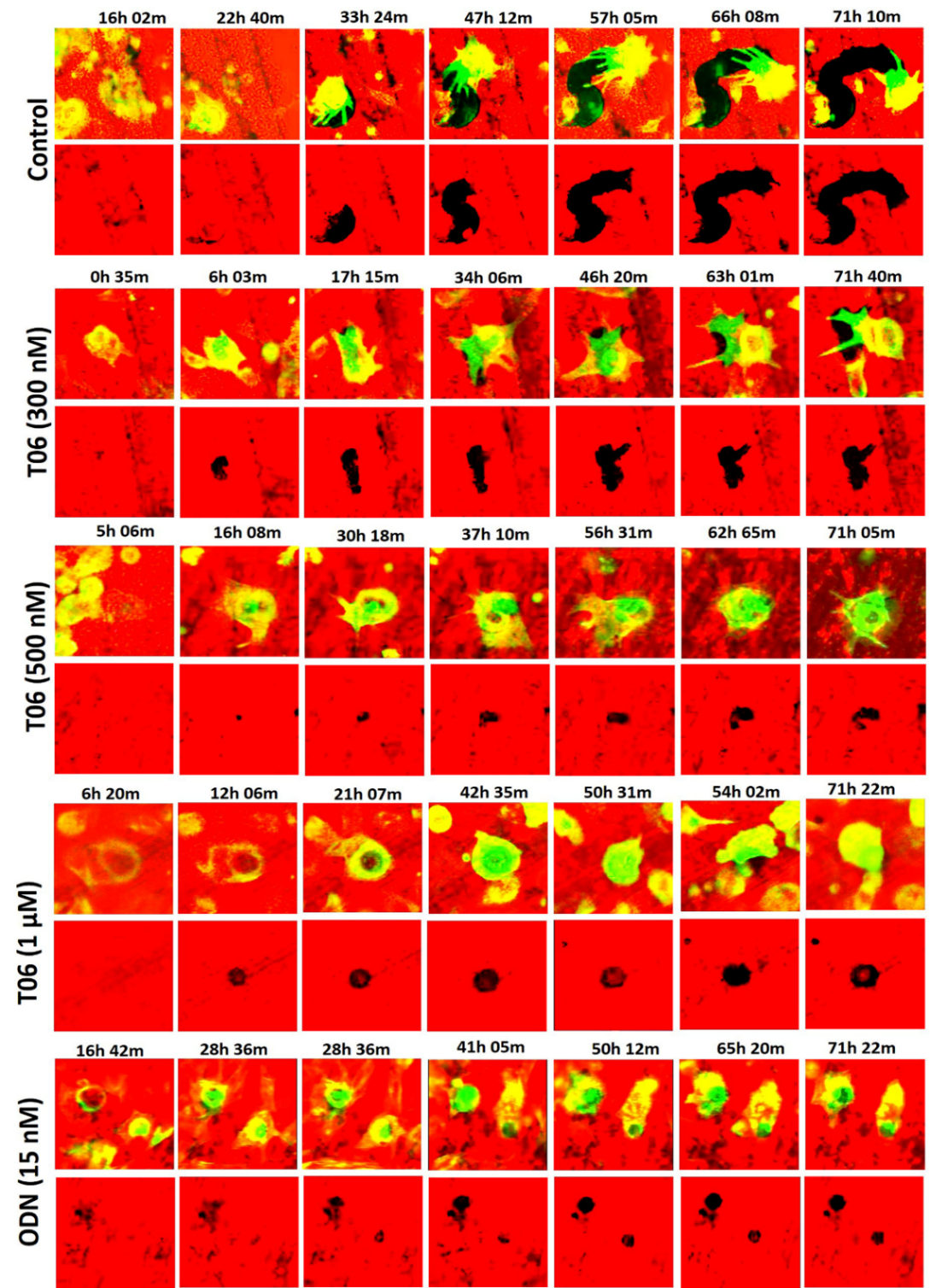


**Supplementary Fig. S4:** **Consecutive images of OCs involved in forming resorption excavation during 72 h in typical and slow resorbing mode (stagnating activity) after CatK inhibition by 300 nM T06 (Video 2), 500 nM T06 (Video 3), 1µM T06 (Video 3), and 15 nM ODN (Video 4).** These OCs continue showing the actin signal typical of the resorption mode and move erratically (as indicated by no major removal of rhodamine despite the permanence of a SZ/RB, which they then orient in multiple successive directions).


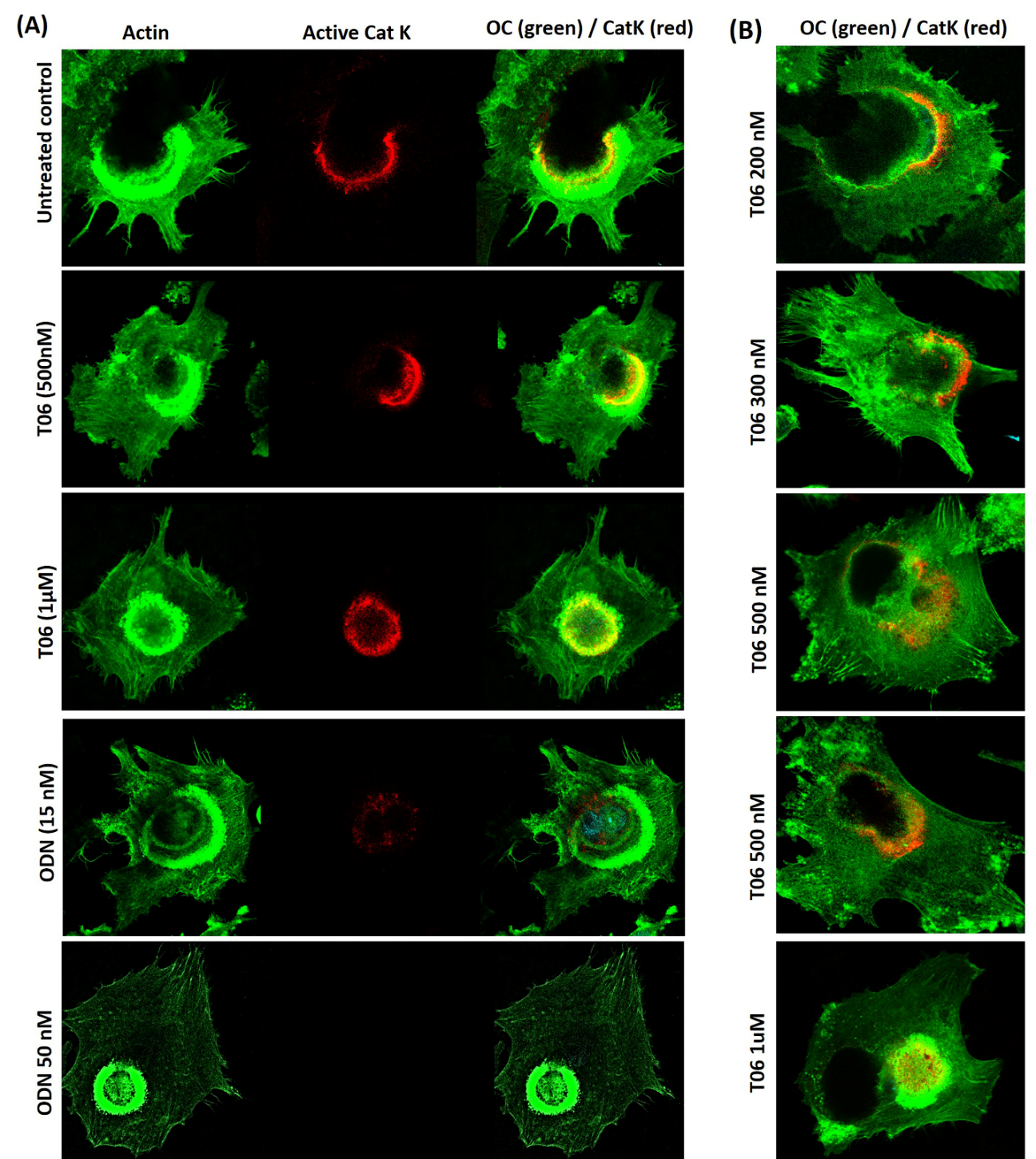


**Supplementary Fig. S5**: **Demonstration of CatK activity in resorbing OCs by an** **activity-based probe.** (A) Each picture is a 3D z-projection of a single OC in trench, deep, and shallow pit formation mode. OCs are visualized in green (actin) and CatK activity is visualized in red. An ectosteric inhibitor, T06, specifically blocking the collagenase activity of CatK by preventing protease oligomerization did not prevent labeling of CatK by the activity-based probe. ODN is an active site-directed inhibitor and thus blocks labeling. (B) Representative pattern of OCs and CatK expression in T06-inhibited OCs at different concentration after 72 h resorption (3D z-projections). Representative pictures of each condition showed the same OCs engaged in making multiple adjacent resorption events at higher inhibitor concentrations tested. However, OCs in trench-making mode were frequently observed when treated with lower T06 concentrations.
