# Supplementary material for: Real-time analysis of osteoclast resorption and fusion dynamics in response to bone resorption inhibitors
Source: Sci Rep. 2024 Mar 28;14:7358. doi: 10.1038/s41598-024-57526-9 (PMC10978898; doi:10.1038/s41598-024-57526-9)
Supplement: Supplementary file 10 — Supplementary Video Legends. [file 41598_2024_57526_MOESM10_ESM.docx]

**Video legends**

**Video 1:** Time-lapse video of OCs over 72 h showing the change in their resorption activity with increasing concentrations of CatK inhibitors T06 (200 nM, 300 nM, 500 nM, and 1 µM) and ODN (15 nM and 50 nM). OCs are shown in green and bone surface (collagen) in red. Resorption by OCs over the bone surface is shown by black imprints (trenches and pits).

**Video 2:** Time-lapse video showing the typical resorption behaviour of OCs in trench and pit mode during 72 h ofobservation. The interruption in resorption caused by CatK inhibition (T06 300 nM) is interpreted clearly. OCs are shown in green and bone surface (collagen) in red, excavation is shown by black imprints.

**Video 3:** CatK inhibition by ectosteric inhibitor T06 (500 nM and 1 µM) induces two prominent behaviours: (1) multiple resorption episodes resulting in adjacent pits (~75%) and (2) an atypical behaviour characterized by slower/decelerating erosion and erratic displacements at the same excavation, suggesting stagnating activity of OCs (~25%). OCs are shown in green and bone surface (collagen) in red, with excavation shown by black imprints.

**Video 4:** CatK inhibition by active site-directed inhibitor ODN (15 nM and 50 nM) induces two prominent behaviours: (1) multiple resorption episodes (~75%) and (2) stagnating activity of OCs (~25%). OCs are shown in green and bone surface (collagen) in red, with excavation shown by black imprints.

**Video 5:** Time-lapse videos showing fusion of OCs in the untreated control and T06 (200 nM, 300 nM, 500 nM), and ODN (15 nM) treated cultures. Fusion of OCs increases with gradual CatK inhibition. However, at complete CatK inhibition in OCs fusion events also normalized. OCs are shown in green and bone surface (collagen) in red, with excavation shown by black imprints. Arrows point at the fusion of OCs from initiation until the end.

**Video 6:** Time-lapse videos showing the fusion of OCs while making trenches, pits transforming into trenches, and during multiple pit formation. An increase in the resorption speed of OCs after fusion was observed in both the absence and presence of CatK inhibitors. OCs are shown in green and bone surface (collagen) in red, with excavation shown by black imprints. Arrows are pointing at the fusion of OCs from initiation until the end.
